# The relationship between common data-based indicators and the welfare of Swiss dairy herds

**DOI:** 10.3389/fvets.2022.991363

**Published:** 2022-10-20

**Authors:** Barbara Lutz, Sibylle Zwygart, Beat Thomann, Dimitri Stucki, Joan-Bryce Burla

**Affiliations:** ^1^Centre for Proper Housing of Ruminants and Pigs, Federal Food Safety and Veterinary Office FSVO, Ettenhausen, Switzerland; ^2^Clinic for Ruminants, Vetsuisse Faculty, University of Bern, Bern, Switzerland; ^3^Vetsuisse Faculty, Veterinary Public Health Institute, University of Bern, Bern, Switzerland; ^4^Centre for Proper Housing of Ruminants and Pigs, Agroscope, Ettenhausen, Switzerland

**Keywords:** herd records, welfare assessment, routine herd data, national database, herd health, animal based measures

## Abstract

The assessment of dairy cow welfare has become increasingly important in recent years. Welfare assessments that use animal-based indicators, which are considered the most direct indicators, are time consuming and therefore not feasible for assessments on a large number of farms. One approach to reducing this effort is the use of data-based indicators (DBIs) calculated from routine herd data. The aim of this study was to explore the relationship between common DBIs and the welfare of 35 dairy herds to evaluate the feasibility of a data-based welfare prediction method. For this purpose, the WelfareQuality^®^ (WQ) protocol was used to assess the welfare of dairy cows on 35 Swiss farms, for each of which 10 commonly used DBIs were calculated from herd data. Spearman's rank correlations were used to investigate the relationship between DBIs and WQ criteria and measurements. The study found only a few statistically weak associations between DBIs and animal welfare, with no associations for measurements or criteria of resting comfort and appropriate behavior. Thus, the multidimensional welfare definition is insufficiently covered, and the present publication does not support the approach of a purely data-based prediction of dairy welfare status at the farm level. Instead, the regular calculation of DBIs that are indicative of isolated animal welfare problems or metrics of animal health could allow monitoring of these specific areas of animal welfare.

## Introduction

In recent years, the valid assessment of farm animal welfare has become increasingly important for a growing number of stakeholders. Farmers benefit from animal welfare assessments for self-control, benchmarking their performance, or as advisory tools (1, 2). Market organizations, retailers, and organic farming associations employ welfare assessments by external auditors to ensure compliance with their welfare standards (3, 4) and federal institutions assess the fulfillment of legal welfare requirements and minimum welfare standards (1, 5). However, animal welfare is a complex, multidimensional concept that includes biological functions, animal behavior, and affective state (6). Because of its complexity, animal welfare itself cannot be measured directly but must be reflected by a variety of indicators that represent its multidimensionality (7). The indicators used for on-farm welfare assessments can be divided into two main types: input- and outcome-based indicators. Resource- and management-based indicators are used to survey the environment and the management that affect the animals (input-based). In contrast, animal-based indicators (ABIs) are collected directly from the animal and can thus indicate how the animal copes with the influencing factors (outcome-based) (1, 8). Therefore, ABIs are considered to reflect welfare more directly, leading to an increasing preference for ABIs over input-based indicators (9). Probably the most comprehensive welfare assessment protocol for various livestock species is the approach developed by the WelfareQuality^®^ (WQ) project (10, 11). The WQ protocol for dairy cows includes 27 indicators that are mostly animal-based and complemented by input-based indicators only in areas where no suitable ABIs are available (12). However, conducting on-farm surveys, especially the assessment of animal-based indicators, is very time-consuming. Approximately 6 h are required to survey a herd of 60 cows using the WQ protocol (13). To survey larger numbers of farms, such as for monitoring animal welfare at the population level, alternatives must be found to allow a quick and cost-effective assessment (14).

Given the challenge of alternative approaches to assessing animal welfare, various attempts have been made to make animal welfare assessments on large numbers of farms more feasible or to shorten the duration of surveys. One way to accomplish this is to integrate routinely collected herd data into animal welfare monitoring, which could replace ABIs (8) or enable data-driven risk screening that could reduce the number of farm visits (15). Herd data collected directly from animals, such as somatic cell counts in milk, can be considered indirect animal-based indicators (16) or data-based welfare indicators (DBIs). Whereas traditional welfare assessments, such as the WQ protocol, usually include only a few DBIs, some research has aimed to predict farm-level welfare status based solely on DBIs (14, 15, 17–19). In this way, a data-based screening should be created that could limit on-farm surveys to high-risk farms.

A precondition for a wide use of DBIs, for example within the framework of national monitoring, is the availability of data of sufficient quality from as many farms as possible (20). This approach seems particularly suitable for dairy farms, as, due to European legislation, a large amount of routine herd data are available. For example, cattle must be individually identifiable, and data on birth, movement, and death must be collected and stored in national databases (21). Furthermore, analyzes of bulk milk delivered for food production must be carried out on a regular basis (22). These data are supplemented by milking records of individual animals obtained monthly by breeding or producer organizations in many countries (23).

In addition to the availability of the data, it is necessary that the DBI is related to the animal welfare of the farm to use a DBI to predict animal welfare (20). Based on the results of previous work, the aim of this study was to investigate the relationship between common data-based indicators and the welfare of Swiss dairy herds in order to assess the potential of data-based indicators to estimate the animal welfare of Swiss dairy herds.. For this purpose, an on-farm survey was conducted on 35 Swiss dairy farms. The animal welfare status was assessed using the WQ's criteria and measurements and subsequently examined for its association with 10 selected DBIs calculated from herd data.

## Materials and methods

### Farms and animals

Farm visits were conducted on Swiss dairy farms from January 2020 to March 2021. To recruit farms, farmers interested in previous studies or recommended by other farmers were contacted by telephone. Thirty-seven farmers agreed to participate and fulfilled the condition of having at least 16 lactating dairy cows at the time of the planned farm visit. The farms were visited once during the winter housing period between January and March (22 farms in 2020, 15 farms in 2021), after the cows had been mainly housed indoors for a minimum of 2 months. Of the 37 farms visited, 35 farms with a complete on-farm welfare assessment delivered valid values and were included in the analyzes.

The mean annual herd size was 47 dairy cows (range 16–136). Twenty farms had a free stall barn, and on 15 farms cows were kept in tie-stalls. Seven farms were run according to certified organic standards (Bio Suisse). All tie-stall farms participated in the Swiss animal welfare program RAUS (24), which requires regular outdoor exercise during winter and pasturing in summer. All loose housing farms participated in the Swiss animal welfare program BTS (24), which requires a comfortable lying area separated from the feeding area. In addition, 18 of the 20 loose housing farms participated in the RAUS program.

### Assessment of farm animal welfare status using the WQ protocol

The welfare status of the herds was surveyed by conducting the entire WQ protocol for dairy cattle (12). All assessments were carried out by the first author, who had previous experience in dairy farming and the handling of cows. The proper application of the WQ was trained in a 3-day course given by an official trainer of the WQ consortium and routinized on three test farms, which were not included in the data analysis.

Farm visits started at the end of morning milking or, alternatively, at morning feeding, when the cows were at the feeding table. All measurements were collected following the guidelines of the WQ protocol. This involved direct observations of the herd, examinations of individual animals and husbandry conditions, and an interview with the farm manager. Additional information was derived from the farm records.

According to the guidelines of the WQ protocol (12), the on-farm measurements were expressed as herd-level prevalence or frequencies on a continuous scale and aggregated into WQ criteria scores ranging from 0 to 100. As the WQ does not currently include a measurement of thermal comfort, this criterion was not considered (see Table 1).

**Table 1:** Overview of WelfareQuality^®^ principles, criteria, and measurements as well as the expressions at herd level used for the analysis [Table modified from (12)].

	**Criteria**	**WQ indicators**	**Measurements/herd-level expressions used for analysis**
Principle: Good feeding	1. Absence of prolonged hunger	Body condition score	% BCS very lean
			% BCS fat^†^
	2. Absence of prolonged thirst	Water provision (number, length of water troughs/bowls)	
		Cleanliness of water points	*
		Water flow	*
		Functioning of water points	*
Principle: Good housing	3. Comfort around resting	Time needed to lie down	Mean time to lie down
		Animals colliding with housing equipment during lying down	% Collisions with stalls
		Animals lying outside the lying area	% Lying outside lying area
		Cleanliness of udder	% Dirty udders
		Cleanliness of flank/upper legs	% Dirty hindquarters
		Cleanliness of lower legs	% Dirty legs
	4. Thermal comfort	No measure developed yet	
	5. Ease of movement	Presence of tethering	*
		Access to outdoor loafing area or pasture	*
	6. Absence of injuries	Lameness	% Not lame^†^
			% Moderately lame
			% Severely lame
		Integument alterations	% Cows without skin alterations^†^
			% Cows with hairless patches
			% Cows with severe skin alterations
Principle: Good health	7. Absence of disease	Coughing	Frequency of coughing (coughs/cow/15 min)
		Nasal discharge	% Nasal discharge
		Ocular discharge	% Ocular discharge
		Hampered respiration	% Hampered respiration
		Diarrhea	% Diarrhea
		Vulvar discharge	% Vulvar discharge
		Milk somatic cell count	% Mastitis
		Mortality	% Mortality
		Dystocia	% Dystocia
		Downer cows	% Downer cows
	8. Absence of pain induced by management procedures	Disbudding/dehorning	*
		Tail docking	*
Principle: Appropriate behavior	9. Expression of social behaviors	Agonistic behaviors	Frequency of head butts (head butts/cow/h)
			Frequency of displacements and other agonistic interactions (interactions/cow/h)
	10. Expression of other behaviors	Access to pasture	*
	11. Good human-animal relationship	Avoidance distance	*
	12. Positive emotional state	Qualitative behavior assessment	*

^†^WQ protocol foresees survey and evaluation at herd level, but not inclusion in calculation of indicators and criteria.

^*^ Exclusively considered as criteria score, individual measurements not taken into account for analyzes.

### Calculation of data-based indicators used as animal welfare indicators

For the present study, DBIs were investigated that have already been used as animal welfare indicators or that are considered to be relevant for this purpose. In addition, the DBIs had to be calculable using data routinely available in Switzerland. To identify the DBIs, results from previous scientific literature (25) were used. These DBIs were supplemented with DBIs that are currently being used in other animal welfare projects or assessments, such as Q-check (26) or AssureWel (27), even if no peer-reviewed reports have yet been published for these projects or assessments. All DBIs fitting the criteria (routinely available, identified in scientific literature, or used in other projects) are listed in Table 2.

**Table 2:** Data-based indicators, and their calculations and data sources used for the present study and reasons for the inclusion in the analyzes.

**Data-based indicators**	**Definition and description**	**Data source**	**Reason for inclusion in the analyses**
Cow mortality (%)	$\frac{Dead\;and\;euthanized\;cows}{Herd\;size\;\left(total\;number\;of\;cows\right)}\;\times 100$	Data on identification and registration, Swiss animal	Use as a data-based animal welfare indicator (12, 26–29)
Culled cows in 0–60 DIM (%)	$\frac{Culled\;cows\;in\;0-60\;DIM\;}{Total\;culled\;cows}\;\times 100$	movement database	Potential indicator of health problems in early lactation (30)
Stillbirths (%)	$\frac{Stillborn,\;euthanized\;and\;dead\;calves\;up\;to\;48\;h}{Total\;number\;of\;calves\;born}\;\times 100$		Use of similar data-based animal welfare indicators (26, 27)
Mean productive lifespan (months)	*Mean timespan between the day of first calving and day of culling of all the cows culled during the 1-year period*		Use as a data-based animal welfare indicator (26, 28)
Cows with SCC < 1,00,000 cells/ml (%)	$\frac{Cows\;with\;SCC<1,00,000\;cells/ml}{Total\;number\;of\;cows\;sampled}\;\times 100$	Monthly milk testing, breeding organizations	Recommended indicator for veterinary herd management (31)
Cows with SCC > 2,00,000 cells/ml (%)	$\frac{Cows\;with\;SCC>2,00,000\;cells/ml}{Total\;number\;of\;cows\;sampled}\;\times 100$		
Cows with SCC > 4,00,000 cells/ml (%)	$\frac{Cows\;with\;SCC>4,00,000\;cells/ml}{Total\;number\;of\;cows\;sampled}\;\times 100$		Use as a data-based animal welfare indicator (12, 26, 28)
Milk FPR < 1. 0 in 0–60 DIM (%)	$\frac{Cows\;with\;FPR<1.0\;in\;0-60\;DIM}{Total\;number\;of\;cows\;in\;0-60\;DIM}\;\times 100$		Use of comparable data-based animal welfare indicators (26, 28)
Milk FPR > 1. 5 in 0–60 DIM (%)	$\frac{Cows\;with\;FPR>1.5\;in\;0-60\;DIM}{Total\;number\;of\;cows\;in\;0-60\;DIM}\;\times 100$		
Mean BMSCC (cells/ml)	*Arithmetic mean of BMSCC*	Routine milk analyses of milk delivered for food production	Use as a data-based animal welfare indicator (29), availability for all milk-supplying farms.

All indicators were calculated for a 1-year period in advance of the welfare assessment.

DIM, days in milk; SCC, milk somatic cell count; FPR, fat-to-protein ratio; BMSCC, bulk milk somatic cell count.

All farm-specific data were obtained with the consent of the farm managers. For each farm, data on cattle identification and registration were obtained from the Swiss animal movement database. Where available, data on bulk milk analysis were obtained from the national milk quality database dbmilch, and data on cow-individual milk analysis were retrieved from the breeding associations. From the data sets, the 10 selected DBIs were calculated for an annual period prior to the farm visit (see Table 2). As two farms did not supply milk for human consumption and were therefore not subjected to mandatory bulk milk analysis, the variable mean number of somatic cells in bulk milk (BMSCC) was calculated for 33 farms. Three farms did not participate in the monthly milk recording of individual cows; thus, DBIs derived from the monthly milk analyzes could only be calculated for 32 farms [cows with SCC < 1,00,000 cells/ml (%), cows with SCC > 2,00,000 cells/ml (%), cows with SCC > 4,00,000 cells/ml (%), cows with milk fat-to-protein ratio < 1.0 in 0–60 days in milk (%), cows with milk fat-to-protein ratio > 1.5 in 0–60 days in milk (%)].

### Statistical methods

All analyzes were performed in R version 3.6.3 (32). Descriptive analyzes included the scores of the criteria except for the criterion *thermal comfort*. Furthermore, for criteria with more than one animal-based measurement specified in the WQ, the individual measurements, aggregated at the herd level were included in the analyzes (in particular for the criteria *absence of prolonged hunger, comfort around resting, absence of injuries, absence of disease*, and *expression of social behaviors)*.This included also measurements that were collected and evaluated at the herd level as specified in the WQ but are not intended to be used in the calculations of indicators and scores (e.g., % fat cows, etc.) (see Table 1). The distribution of WQ measurements, WQ criteria, and calculated DBIs was described using minima, maxima, upper and lower quartiles, means, and medians.

To assess potential associations between DBIs and WQ measurements or criteria, we used Spearman rank correlations on each pair of DBI and either the WQ measurements or criteria. Spearman ranks were chosen because the farm results were not normally distributed in the criteria and measurements. The Spearman rank correlations were corrected for tied values. For the criteria *absence of prolonged thirst, ease of movement, and absence of pain induced by management procedures*, farm results were each grouped in three ranks. These three criteria differ from the other criteria in that their assessment in the WQ is not obtained on a continuous scale. Instead, decision trees were used to compile the measurements into a limited number of possible scores. The limited number of ranks achieved led to the exclusion of the criteria *absence of prolonged thirst, ease of movement, and absence of pain induced by management procedures* from the subsequent analyzes. Furthermore, the farm results for the measurements *% hampered respiration, % nasal discharge, and % collisions with stalls* were grouped on a limited number of different ranks, which led to their exclusion from further analyzes. In total, the relationship between the DBIs and eight criteria scores and 23 measurements were analyzed.

As the number of pairwise comparisons increases the risk of false positive results, the obtained *p*-values were adjusted using Benjamini and Hochberg's false discovery rate adjustment (33). Because adjusting for false positives inadvertently increases the risk for false negatives, we carefully assessed all associations with an unadjusted *p*-value ≤ 0.05 based on plausibility, the correlation coefficient, and the unadjusted and adjusted *p*-value.

## Results

### Results of the welfare assessment and the calculation of data-based indicators

**Figure 1:**
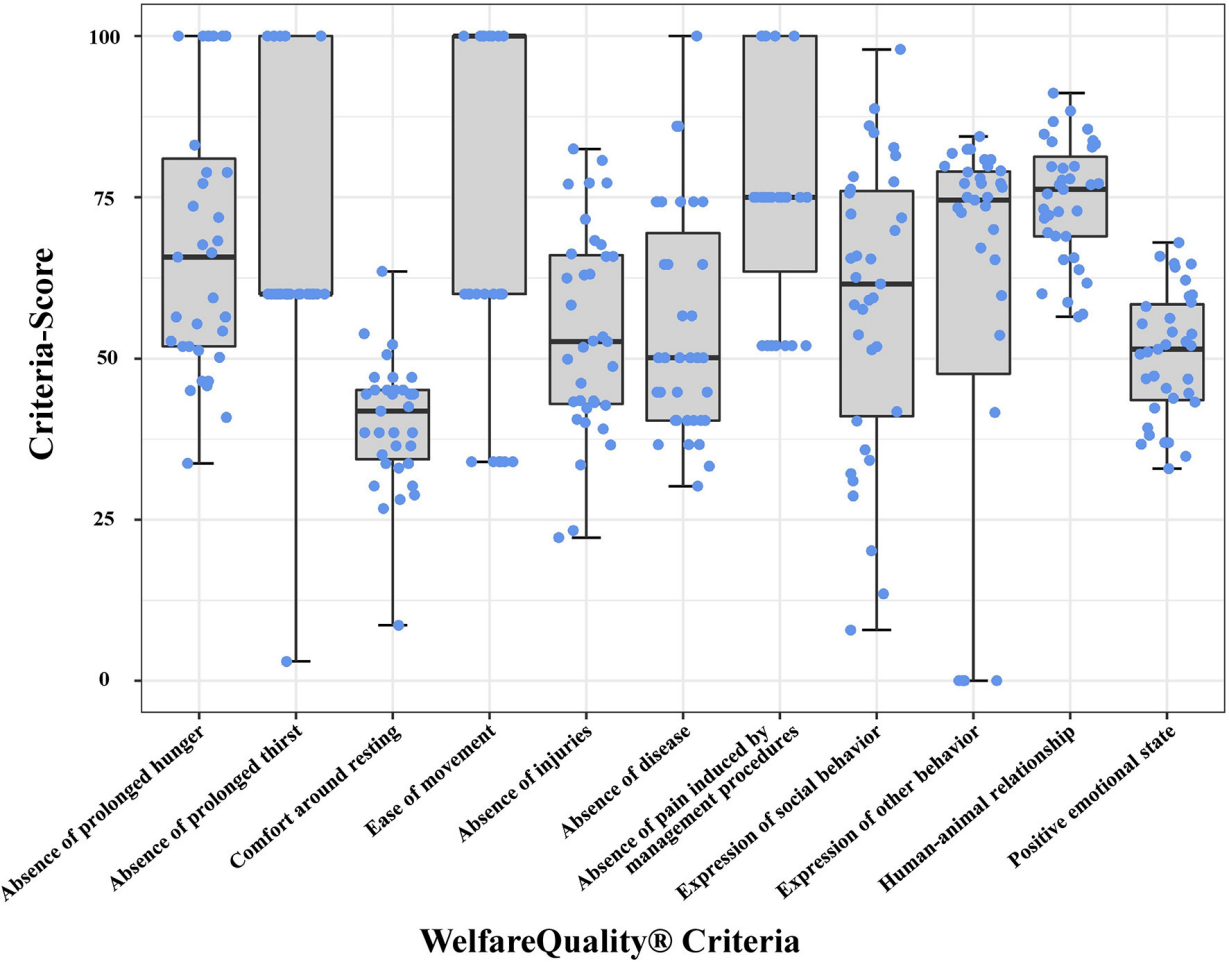
Boxplots and jitter plots representing the WQ scores of 35 analyzed Swiss dairy farms in 11 calculable WQ criteria. Boxes represent the distribution of farm scores, whiskers display the range between the lowest and the highest criteria score observed and blue jitters show the individual farm scores. For the criterion *thermal comfort*, no measurement has yet been assigned in the WQ protocol; for reasons of clarity, a presentation has been omitted.

Descriptive results for farm animal welfare expressed as criteria of the WQ protocol are displayed in Figure 1, while results for the evaluated WQ measurements can be found in Supplementary Table 1. Descriptive statistics for the DBIs as calculated from herd data are shown in Table 3.

**Table 3:** Descriptive statistics from 10 common data-based indicators calculated from 35 Swiss dairy farms for a 1-year period in advance of the welfare assessments.

**Data-based indicators**	**Min**	**1st Quartile**	**Median**	**Mean**	**3rd Quartile**	**Max**	**Farms with lacking data**
Cow mortality (%)	0.00	0.00	2.65	2.51	3.85	10.70	
Culled cows in 0–60 DIM (%)	0.00	0.00	2.65	4.12	4.51	18.94	
Stillbirths (%)	0.00	3.30	6.45	6.43	8.33	18.18	
Mean productive lifespan (months)	791.00	1136.00	1492.00	1498.00	1724.00	2876.00	
Cows with SCC < 1,00,000 cells/ml (%)	30.57	52.13	61.22	58.43	63.40	80.80	3
Cows with SCC > 2,00,000 cells/ml (%)	8.00	14.73	19.23	19.68	24.39	32.18	3
Cows with SCC > 4,00,000 cells/ml (%)	3.09	5.43	7.95	8.49	11.55	16.06	3
Milk FPR < 1.0 in 0–60 DIM (%)	0.00	6.27	11.06	11.93	16.58	29.66	3
Milk FPR > 1.5 in 0–60 DIM (%)	1.30	4.91	11.46	13.51	20.03	35.67	3
Mean BMSCC (cells/ml)	64640.00	108292.00	133792.00	138645.00	161500.00	229815.00	2

DIM, days in milk; SCC, milk somatic cell count; FPR, fat-to-protein ratio; BMSCC, bulk milk somatic cell count.

### Associations between animal welfare and data-based indicators

The initial Spearman rank analysis revealed associations based on uncorrected *p*-values between the DBIs and 7 of 23 analyzed WQ measurements, as well as 2 of 8 analyzed WQ criteria (Table 4). WQ measurements found to be associated with DBIs were predominantly indicators of animal health, as was the criterion *freedom from disease*. Furthermore, the criterion *absence of hunger* and the corresponding measurement *% cows very lean* were associated with the DBI cows with a milk fat-to-protein ratio > 1. 5 in 0–60 DIM (%). Out of the five analyzed measurements of the criterion *comfort around resting*, the *percentage of cows with dirty udders* was associated with the mean productive lifespan. No association could be shown for measurements or criteria related to the principle of *appropriate behavior*.

**Table 4:** Associations between measurements and criteria of the WelfareQuality^®^-Protocol and data-based indicators. Associations with an adjusted *p* < 0.05 are highligted in gray.

**WelfareQuality criteria or measurements**	**Data-based indicators**	***r***	***p*-Value**	***p*.adj**
Absence of prolonged hunger	Milk FPR > 1. 5 in 0–60 DIM (%)	−0.38	0.030	0.638
Absence of disease	Stillbirths (%)	−0.37	0.029	0.638
% BCS very lean	Cows with FPR > 1. 5 in 0–60 DIM (%)	0.38	0.030	0.638
% Dirty udders	Mean productive lifespan (months)	0.38	0.024	0.638
% Not lame	Culled cows in 0–60 DIM (%)	−0.39	0.020	0.638
% Mastitis	Cows with SCC < 1,00,000 cells/ml (%)	−0.57	0.001	0.039
	Cows with SCC > 2,00,000 cells/ml (%)	0.79	< 0.001	< 0.001
	Cows with SCC > 4,00,000 cells/ml (%)	0.75	< 0.001	< 0.001
	Mean BMSCC (cells/ml)	0.40	0.022	0.638
% Mortality	Cow mortality (%)	0.57	< 0.001	0.032
	Cows with SCC < 1,00,000 cells/ml (%)	0.42	0.017	0.638
	Cows with SCC > 2,00,000 cells/ml (%)	−0.36	0.046	0.835
% Dystocia	Cow mortality (%)	0.55	0.001	0.039
% Downer cows	Cows with SCC > 2,00,000 cells/ml (%)	0.37	0.039	0.761
	Cows with SCC > 4,00,000 cells/ml (%)	0.50	0.004	0.190
	Mean BMSCC (cells/ml)	0.38	0.031	0.638
	Stillbirths (%)	0.39	0.020	0.638

DIM, days in milk; SCC, milk somatic cell count; FPR, fat-to-protein ratio; BMSCC, bulk milk somatic cell count.

Correction of the *p*-values to multiple analyzes confirmed five associations with a *p*-value < 0.05 at a high level of confidence. After correction, the associations between the measurement *% mastitis* and the DBIs based on the cow-specific SCC as well as the associations between the WQ measurements *% mortality* and *% dystocia* and the DBI cow mortality % yielded a *p*-value < 0.05.

## Discussion

To identify the potential benefits of DBIs for monitoring herd-level welfare, the aim of the present study was to determine the relationship between 10 commonly used DBIs and animal welfare, expressed in terms of WQ measures and criteria. Overall, the results of the present study suggest that only a few criteria or indicators measured with the WQ are associated with the tested DBIs. The analyzes revealed statistically reliable and at the same time strong associations of the DBIs for only the WQ measurements *% mastitis, % mortality and % dystocia*. The two WQ measurements % mastitis and % mortality are both collected from herd data and are thus already data-based indicators. Therefore, these associations are of limited use for the monitoring of dairy welfare.

In addition, associations shown in the initial Spearman-Rank analyzes could also be valuable for predicting animal welfare status, although most of these associations were eliminated after correction for multiple associations. This comes from the fact that the correction used to adjust for multiple analyzes inadvertently increases the risk of false-negative associations. Hence, associations found in the initial analysis that were not significant after correction of the *p*-values may also be worth further investigation. Among these associations, most were found between DBIs and WQ measurements used as indicators of animal health. Only one association with a measurement of the criterion *comfort around resting* was shown, whereas associations with measurements or criteria of the principle *appropriate behavior* were lacking completely.

One might wonder why the present study showed only a few relationships between DBIs and animal based measurements in comparison to previous work (14, 15, 18, 34). One reason could be the number and selection of DBIs included. For our study, a reduced approach that did not include fertility or milk yield data was used, although those DBIs were found to be associated with animal welfare in other studies (14, 15, 18, 34). These data were omitted, as only the DBIs that were calculable for most Swiss dairy farms and allowing for comparisons between farms were included. The milk yield could have limited the comparability between farms due to the diverse intensity levels of Swiss milk production (e.g., localization of the farm in valley or mountain regions, conventional or organic production, production for drink milk or cheese, the use of dual-purpose or high-yield breeds).

In addition, for both reproduction data and milk yield, the relationship to herd welfare is unclear (35, 36), with a direct link strongly questioned (37). Furthermore, even previous studies examining broad sets of DBIs were unable to predict all criteria of animal welfare. In 2011, a review observed that only a few studies reported relationships between DBIs and measurements of resting comfort and animal behavior (38). On one hand, this was explained by a general lack of studies examining correlations between DBIs and behavior or resting comfort, as was also reported recently (25). On the other hand, the potential of herd data to detect problems in resting comfort and behavior was questioned (38). Indeed, even among the studies that included behavioral parameters, the number of associations between DBIs and animal welfare was low compared to other parameters (14, 15, 34). Hence, the lack of relationships between DBIs and measurements of behavior or comfort of resting might be due to the nature of the DBIs. With the exception of the mean productive lifespan, the DBIs included in the present study are closely related to animal health or describe risk factors for impaired animal health. In contrast, none of the DBIs has a strong direct relationship with animal behavior or resting comfort. Whereas the considered DBIs and the health-related measurements could be linked by common factors (e.g., health management), resting comfort and animal behavior were likely to have no common link with selected DBIs.

Our study was intended as a preliminary investigation to determine relationships between DBIs and animal welfare to estimate the predictive potential of DBIs for animal welfare at the herd level. Regarding the methodology used, one may question why criteria and herd-level measurements were used to express the welfare status of herds rather than the overall score. The overall score was omitted as it results from a multi-step weighted aggregation of measurements, which partly allows the compensation of different welfare aspects (39). However, the weighted and compensating aggregation is questioned in animal welfare research, as it has been shown that the overall score is strongly influenced by only few measurements (11, 40, 41). Furthermore, the weightings, which were determined partly based on expert opinion (39), have not been adjusted to reflect changes in agriculture and changing attitudes toward animal welfare.

Concerning the statistical methodology, the present study investigated univariate relationships between DBIs and welfare measurements and criteria. This approach derives from current efforts to routinely evaluate a range of DBIs that are not aggregated into predictive models (26). Furthermore, the approach was chosen to facilitate comparison with previous studies on DBIs that also initially analyzed univariate associations between DBIs and animal-based measures (14, 15, 18, 34). It should be noted that, based on the information provided, none of these studies adjusted the univariate associations for the presence of multiple analyzes. The results of the present study suggest that univariate relationships between DBIs and welfare measurements and criteria obtained without correction for multiple testing should be interpreted with caution.

It is clear that replicating the present study with more farms, possibly targeting farms with suspected good or poorer animal welfare status or a random selection of farms, would increase the reliability of the results. Nonetheless, in connection with the results from the literature, conclusions can be drawn for the predictive potential of DBIs. The predominantly statistically weak associations of the tested DBIs with only a few measurements of WQ indicate that the tested DBIs are not sufficient to comprehensively predict animal welfare. Given the inadequate coverage of behavioral measurements and indicators of resting comfort, it is questionable whether additional DBIs could complete the predictability of dairy herd welfare status as described by the WQ. This is in line with previous studies which—despite finding a number of associations between DBI and welfare measurements—concluded that associations found were limited (34) and DBIs could only identify problem herds with moderate accuracy rather than estimate the welfare status on the farms (14, 15, 18). Since both the currently used animal welfare definitions (6) and the Swiss Animal Welfare Act (42) require a multidimensional definition including species-appropriate behavior and adequate husbandry, we doubt the applicability of DBIs to predict animal welfare in its multidimensionality in the near future.

Nevertheless, DBIs should not be generally considered inappropriate for the monitoring of dairy cow welfare at the herd level. All 10 variables investigated in the present study are used in veterinary medicine or herd monitoring to gain insights into herd-level animal health status (43, 44). Moreover, increased cow mortality (45) or a high stillbirth rate (46) can themselves be considered animal welfare issues. For example, applying current alarm thresholds to the DBI % cow mortality [4–5% cows (47, 48)] would classify about 14% of the study farms as at-risk for the welfare problem of high cow mortality. Thus, the more welfare issues that can be captured using data, the more direct DBIs could be applied to identify farms at risk. However, since only a limited number of animal welfare issues can currently be monitored directly by data screening, it must be clear that good performance in these parameters does not necessarily reflect a sufficient herd welfare status.

## Conclusions

This study demonstrated few associations between DBIs and animal welfare as measured by the WQ protocol. The associations shown for DBIs were predominantly statistically weak and emerged for a limited number of criteria and herd-level measurements of the WQ, with no associations identified with resting comfort or appropriate behavior. Thus, as DBIs were not able to adequately reflect the multidimensionality of animal welfare, the study suggests that the potential of DBIs is to provide information on specific welfare aspects rather than to provide a comprehensive predictive tool for dairy welfare status at the herd level.

## Data availability statement

The raw data supporting the conclusions of this article will be made available by the authors, without undue reservation.

## Ethics statement

The animal study was reviewed and approved by Cantonal Veterinary Office Thurgau and the Ethical committee for animal experiments of the Cantonal Veterinary Office Zurich. Written informed consent was obtained from the owners for the participation of their animals in this study.

## Author contributions

BT and J-BB: conceptualization and funding acquisition and funding acquisition. BL: investigation and writing—original draft preparation. BL and DS: formal analysis. BT, J-BB, DS, and SZ: review and editing. J-BB: supervision. All authors have read and agreed to the submitted version of the manuscript.

## Funding

This research was funded by the Federal Food Safety and Veterinary Office (FSVO) and the Federal Office for Agriculture (FOAG); project number: 1.18.14TG.

## Conflict of interest

The authors declare that the research was conducted in the absence of any commercial or financial relationships that could be construed as a potential conflict of interest.

## Publisher's note

All claims expressed in this article are solely those of the authors and do not necessarily represent those of their affiliated organizations, or those of the publisher, the editors and the reviewers. Any product that may be evaluated in this article, or claim that may be made by its manufacturer, is not guaranteed or endorsed by the publisher.
