# Supplementary material for: The relationship between common data-based indicators and the welfare of Swiss dairy herds
Source: Front Vet Sci. 2022 Oct 20;9:991363. doi: 10.3389/fvets.2022.991363 (PMC9632483; doi:10.3389/fvets.2022.991363)
Supplement: Supplementary file 1 [file Table_1.DOCX]

Supplementary Material

**Supplementary Table 1**. Descriptive statistics of WelfareQuality®-Measurements assessed on 35 Swiss dairy farms

| **WQ measurement** | **Min** | **1^st^ Quartile** | **Median** | **Mean** | **3^rd^ Quartile** | **Max** |
| --- | --- | --- | --- | --- | --- | --- |
| % BCS very lean | 0.00 | 2.54 | 5.13 | 5.52 | 8.33 | 16.67 |
| % BCS fat | 0.00 | 1.07 | 5.71 | 6.29 | 8.14 | 30.77 |
| Mean time to lie down (s) | 4.44 | 4.80 | 5.22 | 5.28 | 5.61 | 6.80 |
| % collisions with stalls | 0.00 | 0.00 | 0.00 | 3.13 | 0.00 | 33.33 |
| % lying outside lying area | 0.00 | 9.56 | 20.24 | 25.09 | 38.09 | 62.71 |
| % dirty udders | 2.94 | 10.49 | 30.43 | 29.80 | 42.77 | 79.49 |
| % dirty hindquarters | 0.00 | 3.23 | 8.33 | 13.73 | 14.90 | 84.00 |
| % dirty legs | 0.00 | 14.59 | 40.82 | 41.57 | 67.19 | 84.00 |
| % not lame | 41.03 | 73.63 | 82.98 | 79.82 | 90.31 | 96.67 |
| % moderately lame | 0.00 | 0.00 | 10.20 | 11.23 | 17.80 | 33.33 |
| % severely lame | 0.00 | 3.71 | 7.41 | 8.96 | 12.01 | 47.37 |
| % cows without skin alterations | 4.35 | 38.94 | 62.79 | 56.30 | 74.41 | 90.00 |
| % cows with hairless patches | 2.44 | 19.68 | 34.69 | 35.25 | 50.00 | 82.61 |
| % cows with severe alterations | 0.00 | 4.95 | 7.69 | 8.45 | 11.81 | 23.68 |
| Frequency of coughing (coughs/cow/15min) | 0.03 | 0.07 | 0.11 | 0.13 | 0.17 | 0.66 |
| % nasal discharge | 0.00 | 0.00 | 0.00 | 0.74 | 0.00 | 8.89 |
| % ocular discharge | 0.00 | 0.00 | 0.00 | 1.10 | 2.09 | 6.67 |
| % hampered respiration | 0.00 | 0.00 | 0.00 | 0.08 | 0.00 | 2.78 |
| % diarrhoea | 0.00 | 0.00 | 2.56 | 2.27 | 3.33 | 8.89 |
| % vulvar discharge | 0.00 | 0.00 | 0.00 | 1.41 | 2.86 | 8.89 |
| % Mastitis | 0.00 | 6.01 | 8.18 | 7.85 | 11.15 | 15.22 |
| % mortality | 0.00 | 1.29 | 3.11 | 3.23 | 4.75 | 10.75 |
| % dystocia | 0.00 | 2.58 | 5.26 | 6.58 | 7.63 | 44.94 |
| % downer cows | 0.00 | 0.00 | 1.63 | 2.39 | 3.76 | 16.59 |
| Frequency of head butts (head butts/cow/h) | 0.10 | 0.82 | 1.15 | 1.39 | 1.64 | 3.49 |
| Frequency of displacements and other agonistic interactions (interactions/cow/h) | 0.00 | 0.10 | 0.30 | 0.40 | 0.61 | 1.83 |

BCS = Body condition score; SCC = Milk somatic cell count
